# Fascin enhances the vulnerability of breast cancer to erastin-induced ferroptosis

**DOI:** 10.1038/s41419-022-04579-1

**Published:** 2022-02-14

**Authors:** Cong Chen, Bojian Xie, Zhaoqing Li, Lini Chen, Yongxia Chen, Jichun Zhou, Siwei Ju, Yulu Zhou, Xun Zhang, Wenying Zhuo, Jingjing Yang, Misha Mao, Ling Xu, Linbo Wang

**Affiliations:** 1 Department of Surgical Oncology, Sir Run Run Shaw Hospital, Zhejiang University School of Medicine, Hangzhou, China; 2 Biomedical Research Center and Key Laboratory of Biotherapy of Zhejiang Province, Hangzhou, China; 3 Department of Surgical Oncology, Taizhou Hospital, Wenzhou Medical University, Taizhou, China

**Keywords:** Cell death, Breast cancer

## Abstract

Ferroptosis, which is characterized by intracellular iron accumulation and lipid peroxidation, is a newly described form of regulated cell death that may play a key role in tumour suppression. In the present study, we investigated the expression profiles and biological effects of fascin actin-bundling protein 1 (Fascin, gene name *FSCN1*) in breast cancer. In addition, bioinformatics analysis of the TCGA cancer database and gain- and loss-of-function studies showed that Fascin enhances sensitivity to erastin-induced ferroptosis. Mechanistically, Fascin directly interacts with cysteine/glutamate transporter (xCT, gene name *SLC7A11*) and decreases its stability via the ubiquitin-mediated proteasome degradation pathway. Furthermore, we observed that Fascin is substantially upregulated in tamoxifen-resistant breast cancer cell lines, and drug-resistant cells were also more vulnerable to erastin-induced ferroptosis. Taken together, our findings reveal a previously unidentified role of Fascin in ferroptosis by regulating xCT. Thus, ferroptosis activation in breast cancer with high Fascin level may serve as a potential treatment.

## Introduction

Breast cancer is the leading cause of cancer morbidity and mortality in women around the world [1]. According to the indicators oestrogen receptor (ER), progesterone receptor (PR), human epidermal growth factor receptor-2 (HER2), and Ki67, breast cancer is subdivided into luminal A, luminal B, HER2-positive, and triple-negative breast cancer (TNBC). Due to tumour heterogeneity, the biological behaviour, treatment, and prognosis of different breast cancer subtypes are significantly different [2]. Therefore, identifying effective therapeutic strategies and targets for different subtypes of breast cancer is a major focus of clinical research, especially for TNBC.

Ferroptosis, which is characterized by excessive lipid peroxide accumulation, is a newly discovered form of regulated cell death that was defined in 2012 [3]. In terms of morphology, genetics, metabolism, and molecular biology, ferroptosis is significantly different from other forms of cell death, such as apoptosis, autophagy, and necrosis. Erastin functions as a classic inducer of ferroptosis by suppressing cystine/glutamate antiporters, resulting in cellular cystine uptake inhibition and glutathione (GSH) depletion, which ultimately results in the accumulation of lethal lipid reactive oxygen species (ROS) [4]. Recent studies have confirmed that some types of tumour cells can be killed by inducing ferroptosis, including hepatocellular carcinoma [5], lung cancer [6], and gastric cancer [7]. In particular, cancer cells that are resistant to conventional treatments or have a mesenchymal trait may be more vulnerable to ferroptosis [8], indicating a specific and novel therapeutic strategy for cancer research.

The cystine/glutamate antiporter system xc(−), which is composed of a light chain (xCT, gene name *SLC7A11*) and a heavy chain (4F2hc, gene name *SLC3A2*), is a cystine/glutamate transporter that pumps cystine in exchange for intracellular glutamate at a 1:1 ratio [9]. Therefore, system xc(−) plays a pivotal role in promoting intracellular cystine uptake, GSH biosynthesis, and ferroptosis resistance. As xCT is the primary functional subunit of system xc(−), elucidating the underlying mechanisms of xCT regulation in ferroptosis has been a focus of current research. Previous studies have identified a number of regulators of xCT, such as NRF2 [10], ATF4 [11], and OTUB1 [12]. However, our knowledge of xCT regulation remains relatively rudimentary, especially at the posttranscriptional level [13]. Thus, potential targets to suppress xCT in cancer therapy should be further elucidated.

Fascin actin-bundling protein 1 (Fascin, gene name *FSCN1*) supports a variety of cellular structures, including filopodia, microspikes, and other actin-based protrusions underneath the plasma membrane, through its canonical actin-bundling function. Thus, Fascin may functionally contribute to cell motility, invasion, and adhesion [14]. Clinically, aberrant Fascin level is consistently correlated with tumour metastasis and poor prognosis [15–18]. In addition to the classic functions of bundling with actin, Fascin also has nonclassical functions in cancer cells that have rarely been investigated until recently. For instance, Fascin can maintain cancer stemness [19], regulate gene transcription [20] and signalling pathways [21, 22], and interact with several proteins [23].

In the present study, we assessed the expression profiles of Fascin in specific subtypes of breast cancer as well as its function in cancer metastasis. In addition, for the first time, we demonstrate that Fascin interacts with xCT and predisposes cells to ferroptosis by promoting xCT degradation.

## Materials and methods

### Patient specimens

We retrospectively analyzed all consecutive TNBC patients (*n* = 76) from 2010 to 2012 with a pathologically invasive ductal carcinoma. All specimens were obtained from untreated patients who were undergoing primary surgical treatment at the Department of Surgical Oncology, Sir Run Run Shaw Hospital, Zhejiang University School of Medicine.

### Cell culture

Human breast cancer cell lines (HCC1937, HS578T, MDAMB231, BT549, MCF7, T47D, ZR751, MDAMB453, SKBR3, and BT474) were purchased from the Chinese Academy of Sciences (Shanghai, China) and characterized by STR analysis. Tamoxifen-resistant cells (MCF7/TAMR) were established by culturing MCF7 cells in medium supplemented with 1 μM tamoxifen citrate salt (Sigma-Aldrich, T9262-1G) over 6 months, as previously described [24]. Cells were cultured in medium containing 10% foetal bovine serum (FBS) at 37 °C under an atmosphere with 5% CO2.

### Antibodies and chemicals

The following antibodies were used in the present study at the indicated dilution for WB analysis, IP, IHC: Fascin (sc-21743, Santa Cruz; 1:1000 for WB analysis, 2 µg per 500 µg of total protein for IP, and 1:400 for IHC), xCT (12691S, CST; 1:1000 for WB analysis and 1:50 for IP), xCT (ab175186, Abcam; 1:200 for IHC), 4HNE (ab46545, Abcam; 1:200 for IHC), E-cadherin (3195S, CST;1:1000 for WB analysis), N-cadherin (13116S, CST;1:1000 for WB analysis), Vimentin (5741S, CST;1:1000 for WB analysis), ZEB1 (3396S, CST;1:1000 for WB analysis), Snail (3879S, CST;1:1000 for WB analysis), Slug (9585S, CST;1:1000 for WB analysis), GPX4 (ab125066, Abcam; 1:1000 for WB analysis), TFRC (ab214039, Abcam; 1:1000 for WB analysis), ACSL4 (sc-271800, Santa Cruz; 1:1000 for WB analysis), ub (sc-8071, Santa Cruz; 1:500 for WB analysis), β-actin (sc-47778, Santa Cruz; 1:2000 for WB analysis), and GAPDH (sc-47724, Santa Cruz; 1:2000 for WB analysis).

The chemicals erastin (S7242), N-acetylcysteine (S1623), ferrostatin-1 (S7243), liproxstatin-1 (S7699), Z-VAD-FMK (S7023), MG132 (S2619), cycloheximide (CHX) (S7418) and chloroquine (CQ) (S6999) were purchased from Selleck. 3-Methyladenine (3-MA) (189490) was obtained from Sigma Aldrich, and NP-G2-044 (HY-125506) was purchased from MedChemExpress (MCE).

### Wound‐healing assays

Cells (5 × 10^5^) were seeded into six‐well plates and incubated until reaching 80–90% confluence. Scratch wounds were generated using a pipette tip 48 h after transfection. Then, the cells were washed with PBS twice and resuspended in complete medium. The scratch was observed under a phase-contrast microscope at the time of wound generation (0 h) and again after 24 h. The gap width was measured using ImageJ.

### Transwell migration and invasion assays

For the invasion assays, the transwell chambers were pre-coated with Matrigel (Corning Costar, Cambridge, MA, USA). For the transwell migration assays, cells were directly plated in upper chamber. A total of 1 × 10^5^ cells (transfected with a siRNA or plasmid) were resuspended in serum-free medium and added to the upper chamber of a Transwell, after which 600 μL of medium containing 15% FBS was added to the lower chamber. After 24 h of incubation, cells attached to the bottom were fixed in 4% paraformaldehyde and stained with a 0.5% crystal violet solution for 20 min. The cells that adhered to the membrane were imaged and counted.

### Cell viability assays

Cell viability was assessed using a Cell Counting Kit-8 (CCK-8) purchased from Ape Bio (K1018). Cells were seeded in a 96-well plate. Then, after treatment, the medium was replaced with 100 μl of fresh medium containing 10 μl of CCK-8 reagent, after which the cells were incubated in a humidified incubator (37 °C, 5% CO2) for 2 h. Subsequently, the absorbance was measured at 450 nm using a Thermo Scientific Spectrophotometer (1510-00712).

### Cell live/dead assays

Cell live/dead was assessed by Calcein-AM/PI double staining using a flow cytometer. Cells were seeded in a 12-well plate 1 day before treatment. Then, after treatment with different reagents for 24 h, the cells were harvested (including floating dead cells) and stained with Calcein-AM/PI Kit (Beyotime, C2015M) at 37 °C for 30 min. The percentage of the live (Calcein-AM positive) and dead cell population (PI positive) was determines using a BD Accuri C6 flow cytometer (BD Biosciences).

### Lipid peroxidation assays

The relative lipid peroxidation level in cells was assessed using C11-BODIPY dye (Invitrogen, D3861). Cells were seeded in six-well plates 1 day before treatment. Then, after treatment with different reagents for 24 h, the culture medium was replaced with 5 μM C11-BODIPY for 30 min. The cells were then harvested, washed twice with PBS, and resuspended in 500 μl PBS before being analyzed by flow cytometry using an Accuri C6 flow cytometer with a 488 nm laser on an FL1 detector. The presented data show the relative lipid ROS levels normalized to the control samples.

### GSH assays

The relative GSH concentration in cell lysates was assessed using a total Glutathione Assay Kit (Beyotime, S0052) according to the manufacturer’s instructions. The total glutathione content was calculated by measuring the OD value at 412 nm.

### Transmission electron microscopy

Cells cultured in a six-well plate were fixed with a solution containing 2.5% glutaraldehyde in PBS (0.1 M, pH 7.0) at 4 °C for more than 4 h. The samples were washed three times in PBS for 15 min at each step, postfixed with 1% OsO4 in PBS for 1–2 h, and then washed three times in PBS for 15 min at each step. Subsequently, the samples were dehydrated with a graded series of ethanol (30, 50, 70, and 80%) for approximately 15 min at each step and then dehydrated with a graded series of acetone (90 and 95%) for approximately 15 min at each step. Finally, the samples were dehydrated twice in absolute acetone for 20 min each. Subsequently, the samples were placed in a 1:1 mixture of absolute acetone and the final Spurr resin mixture for 1 h at room temperature and then transferred to a 1:3 mixture of absolute acetone and the final resin mixture for 3 h and the final Spurr resin mixture overnight. After embedding, ultrathin sectioning, and staining, digital images were obtained with a Hitachi Model H-7650 TEM.

### RNA isolation and quantitative real-time PCR

Total RNA was extracted using TRIzol Reagent (Invitrogen), and the RNA (1 μg) was reverse transcribed with a HiFiScript cDNA Synthesis Kit (CWBIO, CW2569M). Quantitative real-time PCR was performed using UltraSYBR Mixture(CWBIO, CW0957H). The threshold cycle (Ct) values for each gene were normalized to those of GAPDH, and the 2^−ΔΔCt^ method was used for quantitative analysis. Target genes were PCR amplified using the following primers: Q-*xCT*-F (TCTCCAAAGGAGGTTACCTGC) and Q-*xCT*-R (AGACTCCCCTCAGTAAAGTGAC); Q-*Fascin*-F (CACAGGCAAATACTGGACGGT) and Q-*Fascin*-R (CCACCTTGTTATAGTCGCAGAAC); and Q-*GAPDH*-F (TGACTTCAACAGCGACACCCA) and Q-*GAPDH*-R (CACCCTGTTGCTGTAGCCAAA).

### Plasmid and siRNA transfection and lentiviral infection

Fascin-overexpressing (PCDNA3.1-H_FSCN1-EF1) or negative control (PCDNA3.1-MCS-EF1) vectors were designed and commercially synthesized by Genomeditech (Shanghai, China). Short interfering RNAs (siRNAs) targeting Fascin and xCT (siFascin and sixCT) and a scrambled control siRNA were designed and commercially synthesized by Hanbio (Shanghai, China). The target sequences of the Fascin siRNAs were GCAAGUUUGUGACCUCCAA (sequence 1) and GCUCCAGCUAUGACGUCUU (sequence 2), while that of the xCT siRNAs was UGGAGUUAUGCAGCUAAUU. Cultured cells reached 70% confluence were transfected with 2 μg of plasmid in six-well plates for 6–8 h. Plasmids and siRNAs were transfected into cells using Lipofectamine 3000 (Invitrogen) transfection reagents in OPTI-MEM following the manufacturer’s instructions.

To construct Fascin-knockdown and the corresponding negative control cell lines (MDAMB231 shFascin and MDAMB231 shCon, respectively), cells were seeded in six-well plates and incubated for 24 h with lentiviruses (Hanbio, Shanghai, China) carrying shRNA sequences targeting *Fascin* (GCAAGTTTGTGACCTCCAA) or a scramble sequence (TTCTCCGAACGTGTCACGTAA), respectively. Then, 72 h after the medium was renewed, 1 μg/ml of puromycin was used to kill uninfected cells.

### Immunoprecipitation and immunoblotting

For IP, cells were washed twice with ice-cold PBS and lysed in RIPA lysis buffer (Beyotime, P0013D) supplemented with a complete protease inhibitor cocktail (Roche). Adherent cells were scraped off the dish using cell scrapers, after which the cell suspension was incubated at 4 °C for 30 min. The cells were then centrifuged in a microcentrifuge at 4 °C and transferred to a clear tube. Protein A/G agarose beads (Santa Cruz, sc-2003) were added to preclear the samples at 4 °C for 1 h. Then, the samples were centrifuged at 1000 × *g* for 5 min at 4 °C, and the supernatant (cell lysate) was transferred to new tubes for IP with the indicated antibodies. In general, samples were incubated with 2 μg of primary antibody overnight at 4 °C on a rocker platform. The next day, 50 µl of the resuspended volume of protein A/G agarose beads was added, and the samples were incubated for another 2–4 h at 4 °C. Subsequently, immunoprecipitates were collected by centrifugation, and the pellets were washed 4 times with 1.0 ml of RIPA buffer. After a final wash, the supernatant was aspirated and discarded and the pellet was resuspended in 50 µl of 2× electrophoresis sample buffer.

For immunoblotting, protein samples were subjected to sodium dodecyl sulfate-polyacrylamide gel electrophoresis (Bio-Rad) and transferred to polyvinylidene difluoride membranes (Millipore). The membranes were blocked in 0.1% Tween-20 in Tris-buffered saline (TBS) containing 5% skim milk (BD Biosciences) for 1 h at room temperature before being probed with the indicated first antibodies followed by HRP-conjugated secondary antibodies. The protein bands were visualized with an Amersham Imager 600 (GE Healthcare).

### Immunohistochemistry staining

For IHC staining, tissue slides were deparaffinized in xylene and rehydrated in alcohol, after which endogenous peroxidase was deactivated by treatment with 3% H_2_O_2_ for 5 min. Then, antigen retrieval was performed in a microwave with 0.1 M sodium citrate buffer (pH 6.0). Subsequently, the sections were blocked with 5% normal goat serum for 30 min at room temperature and probed with primary antibodies for 1 h at room temperature. Slides were incubated with poly-HRP secondary antibodies for 40 min in the dark at room temperature, after which sections were counterstained with haematoxylin to visualize nuclei. Images were acquired with a polarized light microscope (Nikon, Eclipse 80i), and two independent pathologists in our hospital analyzed the staining results. The staining intensity of Fascin expression was scored as 0 (negative staining), 1 (weak staining), 2 (moderate staining), 3 (strong staining). A score of 1–3 was defined as positive for Fascin expression.

### Tumour xenograft assay

Animal studies were reviewed and approved by the Ethics Committee for Animal Studies of Zhejiang University. To generate murine subcutaneous tumours, 1 × 10^6^ MDAMB231 control or Fascin knockdown cells in 0.2 ml PBS containing 50% Matrigel (Corning) were subcutaneously injected into the right posterior flanks of 4-week-old immunodeficient female nude mice. After one week, the mice were randomly assigned to different treatment groups (each group consisted of 5 mice) and treated with erastin (20 mg per kg intraperitoneally, twice every other day). Tumour sizes were measured every other day, and volumes were calculated using the following formula: 0.5× length × width^2^. After 10 days of treatment, all mice were euthanized, and the tumours were surgically removed. A portion of each tumour was immediately fixed in 10% buffered formalin for immunohistochemistry.

### Quantitative proteomics analysis

MCF7/TAMR and wild-type MCF7 cells were harvested in cell lysis buffer. Then, the samples were sonicated three times on ice using a high-intensity ultrasonic processor (Scientz) in lysis buffer, followed by protein estimation, normalization, trypsin digestion, TMT/iTRAQ labelling, HPLC fractionation, and LC-MS/MS analysis. Proteins were considered to be significantly differentially expressed when the *p*-value was < 0.05 (fold change (FC) < 0.5) or < 0.05 (FC > 2.0). We thank Jingjie PTM BioLab (Hangzhou, China) for technical assistance.

### Public database analysis

RNA-seq expression profiles from breast cancer cohorts of TCGA database were extracted from the University of California, Santa Cruz (UCSC) Xena Browser, and the GES76275 from the GEO database were used in the present study. Kaplan–Meier plotter (https://kmplot.com/analysis/) was used to analyze the relationship between *Fascin* expression and OS, RFS, and DMFS in the breast cancer data, and log-rank p and HR values were simultaneously obtained. Fascin co-expression was statistically analyzed using the Pearson test (http://www.linkedomics.org). Gene set enrichment analysis (GSEA) was also used in LinkedOmics functional modules to perform Gene Ontology (GO) term annotation and KEGG pathway analysis [25]. The rank criterion was a false discovery rate (FDR) < 0.05, minimum number of genes was 10, and 500 simulations were performed.

### Statistical analysis

The characteristics of the two groups of patients (Fascin negative *vs* Fascin positive) were compared using Pearson’s chi-squared test with SPSS, version 22.0 (IBM Corporation, Armonk, NY, USA). All experiments in vitro were repeated three times. The experimental values were determined using GraphPad Prism 8.3.1 and are presented as the means ± S.D. Comparisons between groups were made using one-way ANOVA, followed by the *t* test. In all cases, *p* < 0.05 was considered to indicate a significant difference.

## Results

### Fascin is highly expressed in TNBC and enhances cells metastasis

**Fig. 1:**
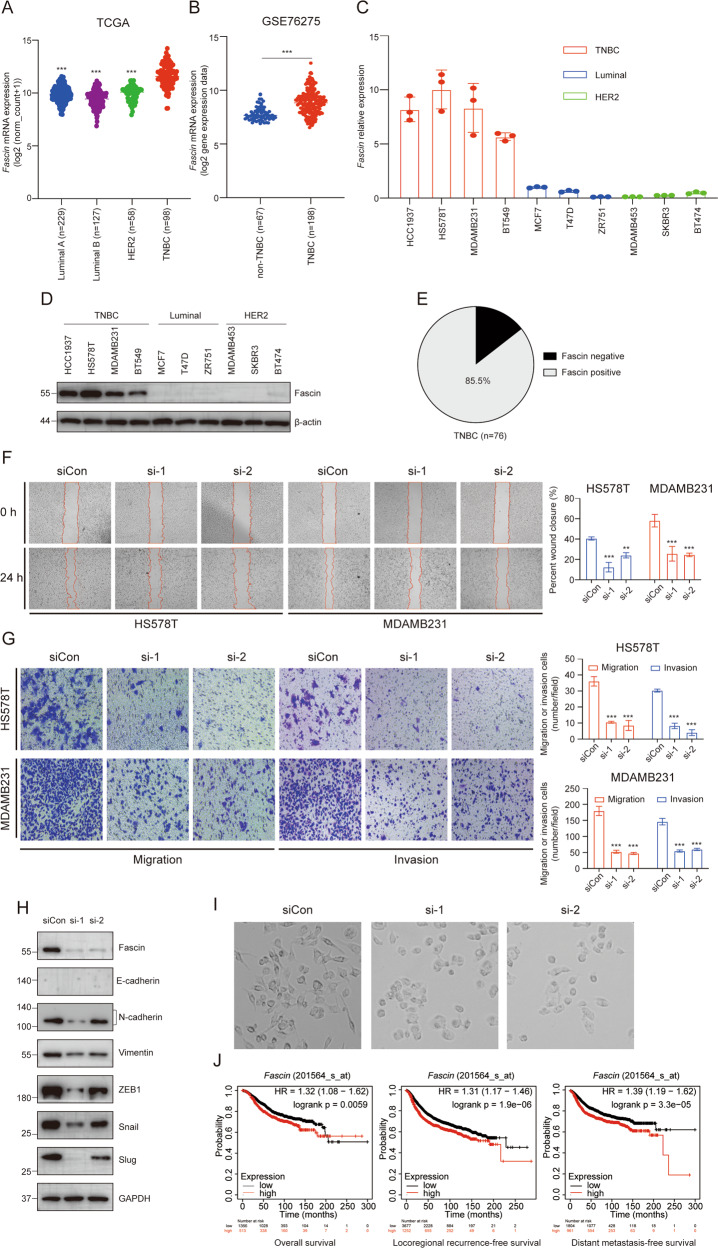
Fascin is highly expressed in TNBC and enhances cell metastasis. **A** *Fascin* expression statuses (unit: log2(norm_count+1)) in TNBC compared to non-TNBC samples were obtained from the TCGA database. **B** *Fascin* expression statuses (unit: log2 gene expression data) in TNBC and non-TNBC samples were obtained from GSE76275. **C** All examined breast cancer cell lines were subjected to qRT-PCR to measure *Fascin* mRNA levels. **D** All examined breast cancer cell lines were subjected to Western blot analysis to measure Fascin protein levels. **E** IHC analysis of Fascin level in TNBC. **F** Wound‐healing assays were conducted in HS578T and MDAMB231 cells transfected with siCon and siFascin. Migration distance was measured at 0 and 24 h after cells were scratched. **G** Transwell migration and matrigel invasion assays were conducted in HS578T and MDAMB231 cells transfected with siCon and siFascin. The cells that adhered to the membrane were imaged and counted. **H** Induction of EMT-related proteins upon Fascin knockdown in MDAMB231 cells. Cell lysates were collected after 72 h transfection. **I** Morphological changes of MDAMB231 cells transfected with siCon and siFascin. **J** The Kaplan–Meier Plotter database (https://kmplot.com/analysis/) was used to show the prognostic value of *Fascin* in breast cancer patients. HR, hazard ratio. **p < 0.01, ***p < 0.001. The data are displayed as the means ± s.d of three independent experiments.

To gain initial insights into Fascin expression patterns in human breast cancer, we screened The Cancer Genome Atlas (TCGA) and Gene Expression Omnibus (GEO) databases. Compared to non-TNBC, *Fascin* expression was potently upregulated in TNBC (Fig. 1A, B). Furthermore, *Fascin* expression was detected in 10 breast cancer cell lines, including luminal, HER2-positive, and TNBC types. *Fascin* was highly expressed in HCC1937, HS578T, MDAMB231, and BT549 (TNBC-derived cell lines) but expressed at relatively low levels in cell lines of other subtypes at both the mRNA (Fig. 1C) and protein levels (Fig. 1D). Additionally, the immunohistochemistry (IHC) results showed that up to 85.5% of TNBC patients were Fascin positive (Fig. 1E), with representative images of Fascin staining for four different extents shown in Fig. S1A. In addition, correlations between Fascin levels and the clinical outcomes of 76 primary TNBC patients are listed in Table 1. Thus, Fascin is generally overexpressed in TNBC cell lines and patients.

**Table 1:** Correlation of Fascin expression and clinicopathological features in TNBC.

	*N* = 76	Fascin negative (*N* = 11)	Fascin positive (*N* = 65)	*p* value
Age (years, means ± SD)		61.0 ± 7.20	50.8 ± 9.44	0.001
Menses status				0.008
Premenopausal	35 (46.1%)	1 (9.1%)	34 (52.3%)	
Postmenopausal	41 (53.9%)	10 (90.9%)	31 (47.7%)	
Tumour size				1
≤2 cm	32 (42.1%)	5 (45.5%)	27 (41.5%)	
>2 cm	44 (57.9%)	6 (54.5%)	38 (58.5%)	
N status				0.782
N0	42 (55.3%)	7 (63.6%)	35 (53.8%)	
N+	34 (44.7%)	4 (36.4%)	30 (46.2%)	
Ki67				0.011
≤30	16 (21.1%)	6 (54.5%)	10 (15.4%)	
>30	60 (78.9%)	5 (45.5%)	55 (84.6%)	
Surgery				0.338
Lumpectomy	27 (35.5%)	2 (18.2%)	25 (38.5%)	
Mastectomy	49 (64.5%)	9 (81.8%)	40 (61.5%)	
Radiotherapy				1
No	33 (43.4%)	5 (45.5%)	28 (43.1%)	
Yes	43 (56.6%)	6 (54.6%)	37 (56.9%)	
Chemotherapy				0.583
No	7 (9.2%)	2 (18.2%)	5 (7.7%)	
Yes	69 (90.8%)	9 (81.8%)	60 (92.3%)	
Recurrence				0.472
Yes	4 (5.3%)	1 (9.1%)	3 (4.6%)	
NO	72 (94.7%)	10 (90.9%)	62 (95.4%)	
Vital status				0.472
Dead	4 (5.3%)	1 (9.1%)	3 (4.6%)	
Alive	72 (94.7%)	10 (90.9%)	62 (95.4%)	

Characteristics of TNBC include high propensity for metastasis and relapse. To elucidate the role of Fascin in TNBC, we performed loss-of-function assays in HS578T and MDAMB231 cell lines. As expected, the wound‐healing, transwell migration, and matrigel invasion assays confirmed that the knockdown of Fascin significantly suppressed TNBC cells migration and invasion, which was consistent with the findings of previous studies [18, 26] (Fig. 1F, G). In addition, immunoblotting results revealed that suppression of Fascin decreased the mesenchymal markers (N-cadherin and Vimentin) expression in MDAMB231 cells (Fig. 1H) and HS578T (Fig. S1B). Furthermore, Fascin-silenced MDAMB231 cells exhibited little mesenchymal morphology (Fig. 1I) compared to those observed in the negative control.

Intriguingly, survival analysis results obtained using Kaplan-Meier plotter (https://kmplot.com/analysis/) indicated that *Fascin* expression is an important indication of overall survival (OS), locoregional recurrence-free survival (RFS), and distant metastasis-free survival (DMFS) in breast cancer (Fig. 1J). Collectively, these findings demonstrate that Fascin may assist in the process of breast cancer metastasis and represent a valuable biomarker for this disease.

### Fascin is a potential novel biomarker of ferroptosis in breast cancer

**Fig. 2:**
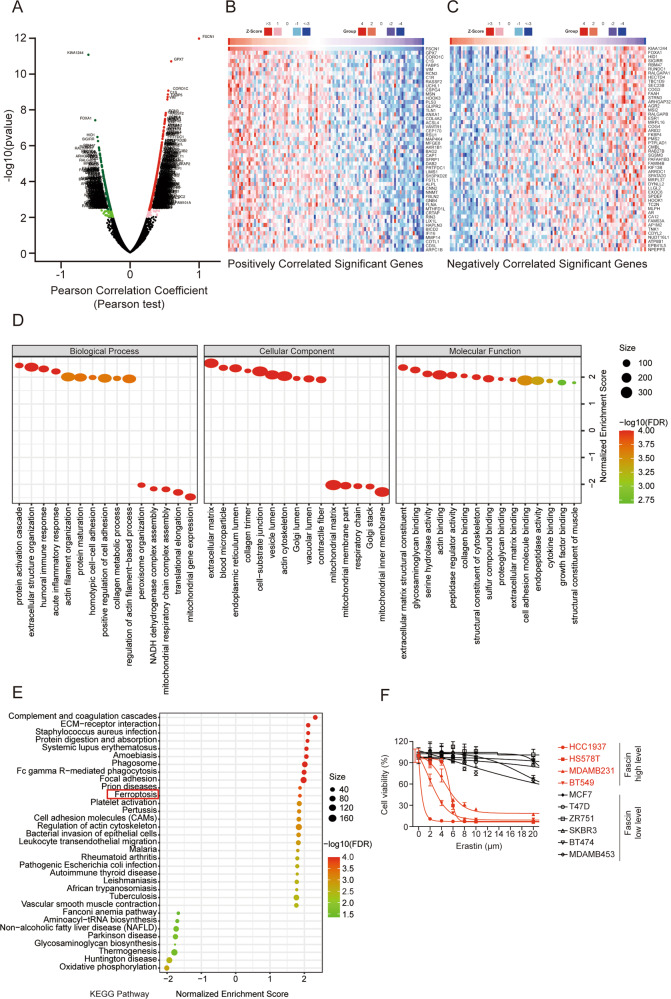
Identification of proteins co-expressed with Fascin in breast cancer using the LinkedOmics database. **A** The volcano plot of Fascin co-expression proteins. Heat maps showing the top 50 proteins positively (**B**) and negatively (**C**) correlated with Fascin. The GO annotation (**D**) and KEGG pathway (**E**) enrichment of proteins co-expressed with Fascin in breast cancer were analyzed using Gene Set Enrichment Analysis (GSEA). **F** Breast cancer cell lines were incubated with the indicated concentrations of erastin for 48 h and then assayed for cell viability. The data are displayed as the means ± s.d of three independent experiments.

To further investigate the signalling pathways regulated by Fascin in breast cancer, we analyzed a number of proteins co-expressed with Fascin in TCGA samples from the LinkedOmics database (http://www.linkedomics.org). A volcano plot showed that 511 proteins were positively associated with Fascin, while 279 proteins were negatively associated with Fascin (Fig. 2A) (FDR < 0.05). The top 50 positively and negatively co-expressed proteins are presented in a heat map (Fig. 2B, C). A strong positive correlation was observed between Fascin and ACSL4 (Pearson correlation = 0.49, *p* = 9.69e−8), while a strong negative correlation was noted between Fascin and ESR1 (Pearson correlation = −0.41, *p* = 1.24e−5).

Next, the enrichment functions of Gene Ontology (GO) and Kyoto Encyclopedia of Genes and Genomes (KEGG) pathways were analyzed using GSEA. The 15 most significant enriched components in each of biological process (BP), cellular component (CC), and molecular function (MF) were presented in Fig. 2D. The primary BP terms identified by GO analysis were protein activation cascade, cell motility processes (actin filament organization, regulation of actin filament, and cell adhesion), and oxidative processes (peroxisome organization, NADH dehydrogenase complex assembly, mitochondrial respiratory chain complex assembly, and mitochondrial gene expression). The top CC term was extracellular matrix. Besides, some mitochondrial components (mitochondrial matrix, mitochondrial membrane part, respiratory chain, and mitochondrial inner membrane) were enriched in the CC terms (Fig. 2D). KEGG pathway analysis showed enrichment in immune pathways (complement and coagulation cascades, systemic lupus erythematosus, and Fc gamma R-mediated phagocytosis) and cell adhesion (ECM-receptor interaction, focal adhesion, and cell adhesion molecules) (Fig. 2E).

Interestingly, we observed that the ferroptosis pathway was enriched in KEGG pathways (Fig. 2E). To assess the association between Fascin levels and ferroptosis sensitivity, we examined 10 breast cancer cell lines divided into two groups (Fascin high *vs* Fascin low levels), and Fascin levels appeared to strongly correlate with the sensitivity of cells to erastin-induced ferroptosis (Fig. 2F).

Previous studies have shown that TNBC cell lines are susceptible to ferroptosis [27], and EMT signalling can also promote ferroptosis [8]. Thus, the high prevalence of Fascin in TNBC, along with its biologic relevance to the EMT and ferroptosis signalling, led us to investigate whether Fascin mediated ferroptosis process.

### Fascin promotes erastin-induced ferroptosis in TNBC

To elucidate the role of erastin-induced cell death, HS578T and MDAMB231 cells were treated with erastin in the absence or presence of several cell death inhibitors. Erastin treatment combined with ferroptosis inhibitors (N-acetylcysteine, ferrostatin-1, and liproxstatin-1) but not inhibitors of apoptosis (Z-VAD-FMK) and autophagy (3-methyladenine) blocked erastin-induced cell death (Fig. S2).

**Fig. 3:**
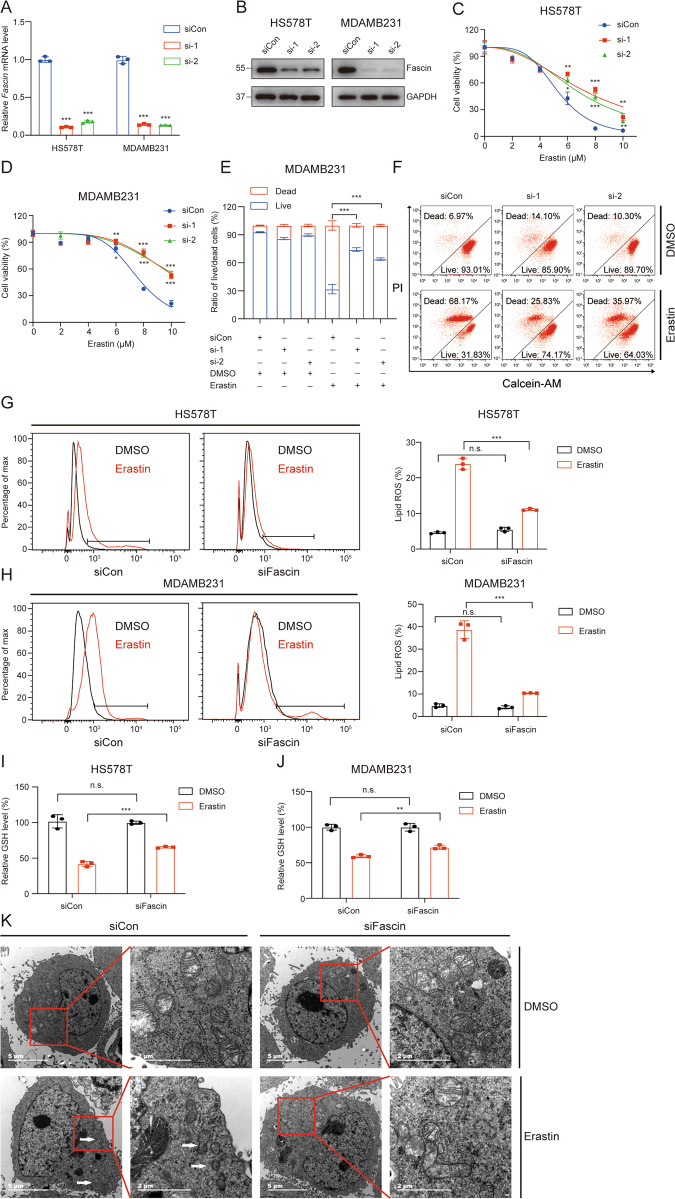
Fascin silencing inhibits ferroptosis sensitivity in the HS578T and MDAMB231 cell lines. The effects of *Fascin* knockdown on mRNA (**A**) and protein (**B**) levels compared to negative controls. HS578T (**C**) and MDAMB231 (**D**) cell lines transfected with *Fascin* siRNAs or siCon were incubated with the indicated concentrations of erastin for 48 h and then assayed for cell viability. **E**, **F** MDAMB231 cell lines transfected with *Fascin* siRNAs or siCon were incubated with erastin (7.5 μM) for 48 h and then assayed to flow cytometry analysis for cell live/dead double staining. HS578T (**G**) and MDAMB231 (**H**) cell lines transfected with *Fascin* siRNAs or siCon were incubated with erastin (5 μM) for 24 h, stained with C11-BODIPY, and then subjected to flow cytometry analysis. HS578T (**I**) and MDAMB231 (**J**) cell lines transfected with *Fascin* siRNAs or siCon were incubated with erastin (0.25 μM) for 48 h and then assayed for GSH levels. **K** MDAMB231 cells transfected with *Fascin* siRNAs or siCon were incubated with erastin (5 μM) for 24 h and then used for TEM analysis. The white arrows show dysmorphic mitochondria (smaller mitochondria, condensed membrane, and decreased cristae). n.s. not significant. *p < 0.05, **p < 0.01, and ***p < 0.001. The data are displayed as the means ± s.d of three independent experiments.

**Fig. 4:**
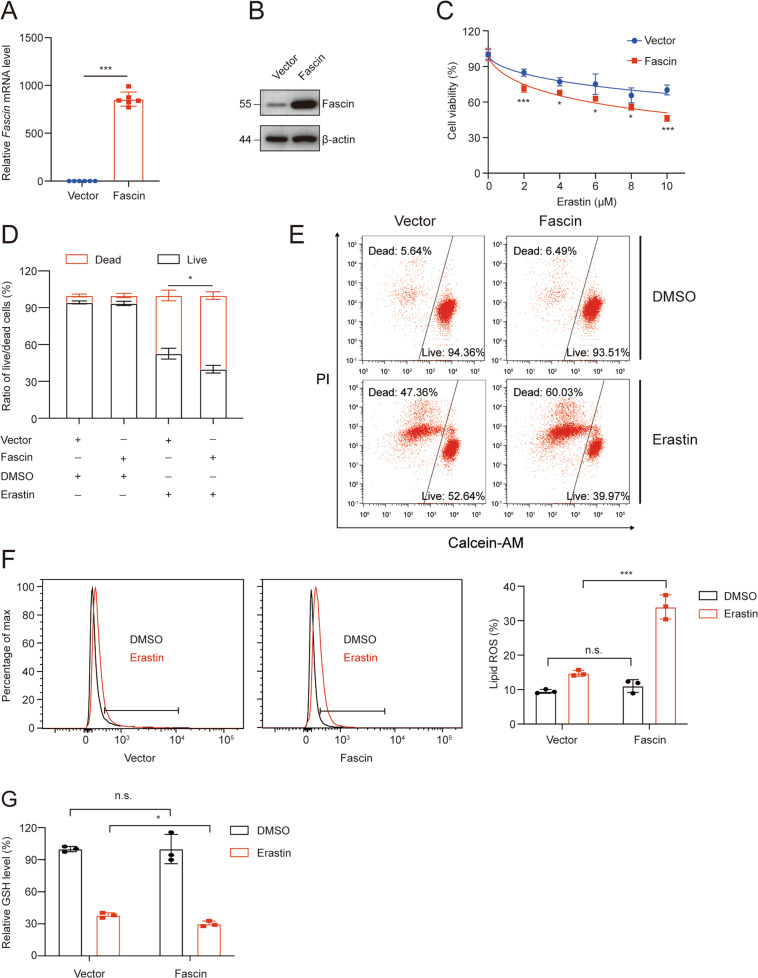
Fascin overexpression promotes ferroptosis sensitivity of MDAMB231 cell lines. The effects of *Fascin* overexpression on mRNA (**A**) and protein (**B**) expression levels compared to the corresponding empty vectors. **C** MDAMB231 cells transfected with an expression vector for *Fascin* or the corresponding empty vectors were incubated with the indicated concentrations of erastin for 48 h and then assayed for cell viability. **D**, **E** MDAMB231 cells transfected with an expression vector for *Fascin* or the corresponding empty vectors were incubated with erastin (5 μM) for 48 h and then assayed to flow cytometry analysis for cell live/dead double staining. **F** MDAMB231 cells transfected with an expression vector for *Fascin* or the corresponding empty vectors were incubated with erastin (2.5 μM) for 24 h, stained with C11-BODIPY, and then subjected to flow cytometry analysis. **G** MDAMB231 cells transfected with an expression vector for *Fascin* or the corresponding empty vectors were incubated with erastin (0.25 μM) for 48 h and then assayed for GSH levels. n.s. not significant. *p < 0.05, and ***p < 0.001. The data are displayed as the means ± s.d of three independent experiments.

To further investigate whether Fascin confers enhanced vulnerability to ferroptosis, we used two different small interfering RNAs (siRNAs) to silence Fascin expression in HS578T and MDAMB231 cell lines. The *Fascin* mRNA and protein levels were significantly reduced compared to those observed in the negative controls (Fig. 3A, B). The CCK-8 assay and cell live/dead double staining results indicated that the knockdown of Fascin confers cells resistant to ferroptosis (Fig. 3C–F). As si-1 and si-2 siRNAs had the same effects (Fig. 3A–F), we arbitrarily selected si-1 for use in subsequent experiments. Consistent with the above findings, suppression of Fascin decreased the erastin-induced elevation of the lipid peroxide levels (Fig. 3G, H) and GSH depletion (Fig. 3I, J). Morphologically, erastin-treated MDAMB231 cells harboured smaller mitochondria that exhibited increased membrane density and with decreased mitochondrial cristae, which is a morphological feature of the mitochondria in ferroptosis. However, these mitochondrial features were prevented in Fascin-silenced MDAMB231 cells treated with erastin (Fig. 3K). As Fascin was moderately expressed in MDAMB231, we overexpressed Fascin in MDAMB231 cells (Fig. 4A, B). Similarly, Fascin overexpression promoted erastin-induced cell death (Fig. 4C–E) in MDAMB231 cells concomitant with increased lipid peroxide levels (Fig. 4F) and GSH depletion (Fig. 4G). Taken together, these results indicate that Fascin can promote erastin-induced ferroptosis in TNBC.

### Fascin interacts with and modulates xCT protein stability

**Fig. 5:**
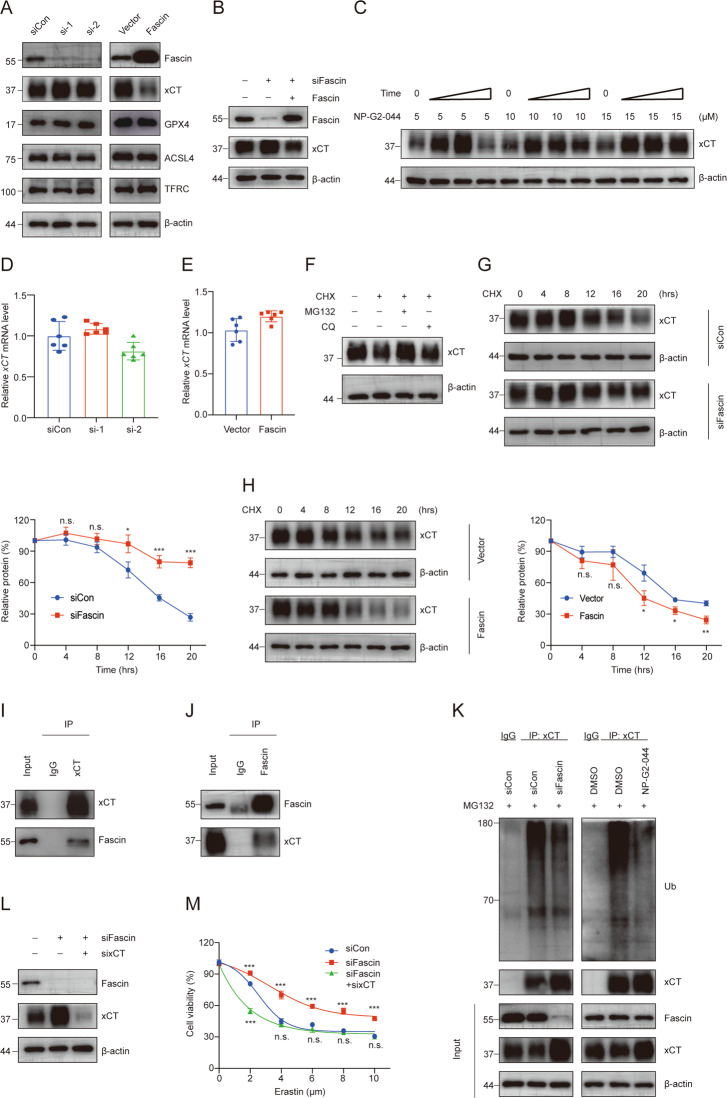
Fascin interacts with xCT and decreases its stability. **A** Induction of ferroptosis-related proteins upon Fascin knockdown and overexpression in MDAMB231 cells. Cell lysates were collected after 72 h of transfection. **B** Western blot analysis showed that Fascin knockdown increased xCT levels, which was reversed by re-expression of Fascin. **C** Western blot analysis of xCT protein levels in MDAMB231 cells treated with NP-G2-044 (5, or 10, 15 μM) for the indicated times (24, 48, or 72 h). *xCT* mRNA was detected in Fascin knockdown (**D**) or Fascin-overexpressing (**E**) cells by quantitative qRT-PCR analysis. **F** Western blot analysis of xCT levels in MDAMB231 cells treated with CHX (100 μg/ml) with or without MG132 (10 μM) and CQ (25 μM) for 16 h. **G**, **H** Fascin knockdown or overexpression cells were treated with 100 μg/ml CHX for the indicated times. Relative xCT protein levels were quantified with ImageJ. **I**, **J** MDAMB231 cell lysates were subjected to immunoprecipitation (IP) with antibodies towards Fascin or xCT or with control immunoglobulin G (IgG). Immunoblot assays were performed using Fascin and xCT antibodies. **K** Immunoprecipitation of cell lysates with Fascin knockdown or incubated with NP-G2-044 (5 μM) cells was performed with xCT antibody, after which lysates were immunoblotted with antibodies against Ub, xCT, Fascin, and β-actin. **L** Immunoblot analysis of Fascin, xCT, and β-actin in MDAMB231 cells transfected with control or *Fascin* siRNAs with or without *xCT* siRNAs for 72 h. **M** MDAMB231 cells transfected with control or *Fascin* siRNAs with or without *xCT* siRNAs were incubated with the indicated concentrations of erastin for 48 h and then assayed for cell viability. n.s. not significant. *p < 0.05, **p < 0.01, and ***p < 0.001. The data are displayed as the means ± s.d of three independent experiments.

To elucidate the underlying mechanisms by which Fascin promotes the vulnerability of cells to ferroptosis, we examined the expression levels of key ferroptosis pathway components. As shown in Fig. 5A, Fascin overexpression in MDAMB231 cell lines decreased the protein level of xCT, whereas Fascin knockdown markedly upregulated xCT levels. To further verify that the effect of Fascin on xCT levels is caused by Fascin knockdown rather than an off-target effect of siRNA, we restored Fascin expression in Fascin knockdown cells. As shown in Fig. 5B, the restoration of Fascin expression reversed the effect of *Fascin* siRNA on xCT level. Similarly, treatment with NP-G2-044, an inhibitor of Fascin activity, increased xCT levels in a dose- and time-dependent manner (Fig. 5C). Collectively, these data demonstrate that Fascin negatively regulates xCT protein levels.

Interestingly, alterations in Fascin levels did not have a significant effect on *xCT* mRNA levels (Fig. 5D, E), indicating that Fascin-mediated changes in xCT may occur at the posttranslational level. Indeed, xCT protein levels were significantly decreased when cells were treated with CHX for 16 h and this effect could be counteracted by the proteasomal inhibitor MG132 but not the lysosomal inhibitor CQ (Fig. 5F), indicating that xCT was regulated by the ubiquitin-proteasome system. In addition, treatment with cycloheximide to block new protein synthesis extended the half-life of the xCT protein following Fascin depletion, while Fascin overexpression shortened the xCT protein half-life (Fig. 5G, H). Moreover, the protein interaction between Fascin and xCT was detected by endogenous immunoprecipitation (IP), confirming that Fascin directly interacts with xCT (Fig. 5I, J). We further assessed the effect of Fascin on xCT ubiquitination, with the data showing that Fascin can promote xCT polyubiquitination (Fig. 5K). To elucidate whether Fascin is involved in the regulation of ferroptosis through xCT, we knocked down Fascin and xCT in MDAMB231 (Fig. 5L, M) and HS578T cells (Fig. S3A, B), and CCK-8 assay results confirmed that xCT knockdown counteracted the Fascin knockdown-mediated resistance to ferroptosis. Taken together, these data indicate that Fascin promotes xCT ubiquitination and contributes to the enhanced vulnerability of cells to ferroptosis.

### Suppression of Fascin inhibition of ferroptosis in vivo

**Fig. 6:**
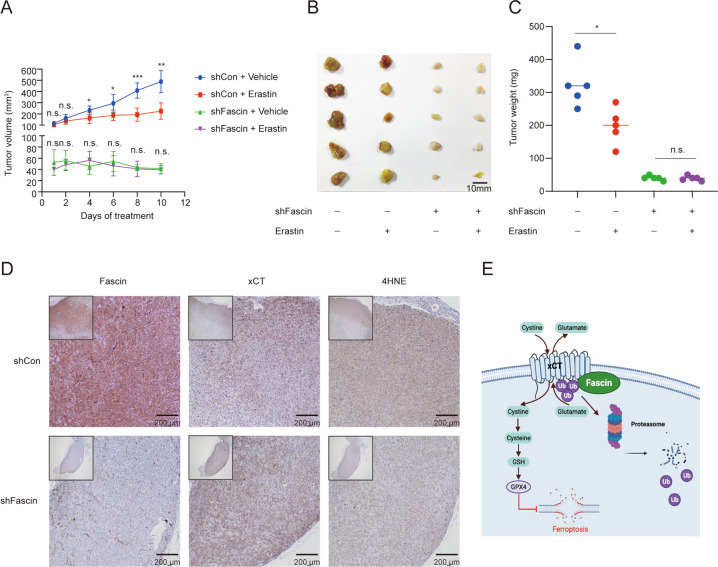
Suppression of Fascin inhibits ferroptosis in vivo. Four-week-old immunodeficient nude mice (five mice per group) were subcutaneously injected with the indicated MDAMB231 cells infected with control or *Fascin* shRNAs (1 × 10^6^ cells per mouse) and treated with erastin (20 mg per kg intraperitoneal, twice every other day) at day seven. **A** Tumour growth curves. **B** Isolated subcutaneous tumours. **C** Tumour weight. **D** Representative images of IHC staining for Fascin, xCT, and 4HNE in the xenografts. **E** Schematic depicting the regulation of xCT by Fascin during ferroptosis. n.s. not significant. *p < 0.05, **p < 0.01, and ***p < 0.001. The data are displayed as the means ± s.d.

To further investigate whether Fascin affects the sensitivity of xenograft tumours to erastin in vivo, MDAMB231 cells with stably inhibited Fascin were injected into the subcutaneous space of the right flanks of mice. On day seven, the mice were treated with or without erastin (20 mg/kg intraperitoneally, twice every other day) for 10 days. Without erastin treatment, the tumours of mice in the control group grew significantly faster and larger than those observed in the mice with Fascin repression (Fig. 6A–C). These data provide support that Fascin promotes tumour proliferation in vivo. However, compared to the control group, Fascin repression made erastin less effective in reducing tumour growth (Fig. 6A–C), further confirming that Fascin suppression can reduce erastin-induced ferroptosis in vivo. In addition, we performed 4-hydroxynonenal (4HNE) IHC analysis to characterize lipid peroxidation levels in tumour xenograft samples. Consistent with the previous findings, IHC analysis indicated a decreased level of 4HNE but an increased level of xCT in the sh-Fascin group (Fig. 6D).

### Activation of ferroptosis in tamoxifen-resistant cells

**Fig. 7:**
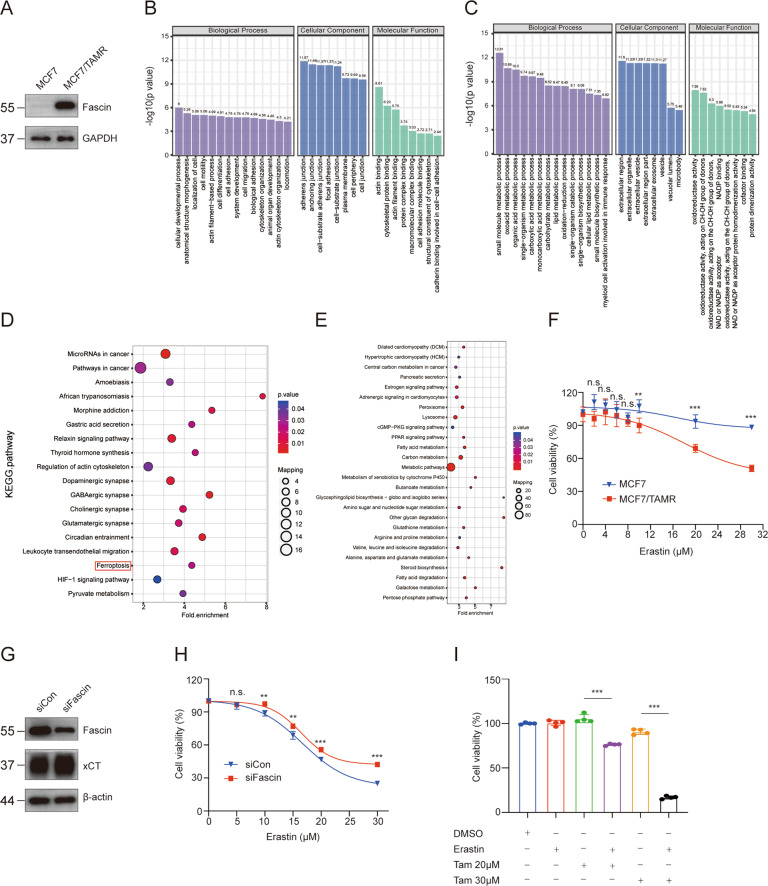
Ferroptosis induction as a novel treatment to suppress tamoxifen-resistant cells. **A** Relative Fascin status in wild-type MCF7 and tamoxifen-resistant MCF7/TAMR cells. **B**–**E** A total of 664 differentially expressed proteins were analyzed for GO annotations and KEGG enrichments. A total of 255 upregulated proteins were analyzed for GO annotations (**B**) and KEGG enrichments (**D**). A total of 409 downregulated proteins were analyzed for GO annotations (**C**) and KEGG enrichments (**E**). **F** MCF7 and MCF7/TAMR cells were incubated with the indicated concentrations of erastin for 48 h and then assayed for cell viability. **G** Western blot analysis showing that Fascin knockdown increases xCT levels in MCF7/TAMR cells. Cell lysates were collected after 72 h transfection. **H** MCF7/TAMR cells transfected with *Fascin* siRNAs or siCon were incubated with the indicated concentrations of erastin for 48 h and then assayed for cell viability. **I** MCF7/TAMR cells were incubated for 48 h with or without 20 or 30 μM 4-OH tamoxifen and in the absence or presence of 5 μM erastin. n.s. not significant. **p < 0.01, and ***p < 0.001. The data are displayed as the means ± s.d of three independent experiments.

We next sought to investigate Fascin as a viable therapeutic biomarker in diseases with a ferroptosis signature. We established a tamoxifen-resistant MCF7 cell line (MCF7/TAMR) by culturing a tamoxifen-sensitive MCF7 cell line in medium supplemented with 1 μM tamoxifen for over 6 months, as previously described [24]. As shown in Fig. S4, MCF7/TAMR cells were much more resistant to tamoxifen than wild-type MCF7 cells, and Western blotting results indicated that Fascin level was relatively higher in MCF7/TAMR cells (Fig. 7A).

To further identify differentially expressed proteins and screen potential treatments for tamoxifen-resistant cells, a quantitative proteomics analysis was performed on MCF7 and MCF7/TAMR cells. The results identified 664 differentially expressed proteins, of which 255 were significantly upregulated and 409 were significantly downregulated in MCF7/TAMR cells compared to MCF7 cells. Subsequently, GO and KEGG pathways were analyzed for the identified differentially expressed proteins. For the upregulated proteins, GO annotation results revealed that these proteins are involved in various cell cytoskeleton remodelling processes, including actin filament-based processes, cell migration, cytoskeleton organization, and actin cytoskeleton organization (Fig. 7B). In addition, KEGG pathway analysis showed that upregulated proteins were enriched in pathways in cancer, regulation of actin cytoskeleton, and ferroptosis (Fig. 7D). Intriguingly, CCK-8 assay results showed that MCF7/TAMR cells were more vulnerable to ferroptosis induction mediated by erastin (Fig. 7F). Further, Fascin suppression upregulated xCT levels and conferred resistance to erastin-induced ferroptosis in MCF7/TAMR cells (Fig. 7G, H), confirming that Fascin is involved in the regulation of ferroptosis in MCF7/TAMR.

Subsequently, treatment of MCF7/TAMR cells with a combination of tamoxifen and erastin showed that the sensitivity of cancer cells to tamoxifen was significantly increased, as confirmed by the cell viability assay results (Fig. 7I), indicating that erastin can act synergistically with tamoxifen to suppress tamoxifen-resistant cells.

## Discussion

TNBC is a subtype of breast cancer that is negative for both ER/PR and HER-2, accounting for approximately 15% of breast cancer patients. TNBC exhibits high heterogeneity, high metastasis, and high recurrence. Even worse, TNBC is insensitive to endocrine therapy and targeted therapy [28]. Thus, the systemic treatment of TNBC is currently a clinical problem and an issue of focus. In this study, the significance of our present discoveries are several folds: First, Fascin is preferentially expressed in TNBC and enhances cells metastasis. Second, we demonstrated that Fascin is a novel regulator of erastin-induced ferroptosis in TNBC by modulating xCT protein stability. Finally, activation of ferroptosis in Fascin-high breast cancer, such as in TNBC and tamoxifen-resistant cancer cells, may be represent an effective treatment strategy.

Previous studies have demonstrated the function of Fascin in cancer proliferation, metastasis, drug resistance, and stemness maintenance [29–31]. In the present study, we demonstrated that Fascin is substantially upregulated in TNBC and is involved in TNBC metastasis, which is consistent with the findings of other studies [19, 32, 33]. Moreover, the non-canonical functions of Fascin are further revealed in our study. To explore the roles of Fascin in breast cancer, we performed bioinformatics analysis using the TCGA breast cancer dataset and observed that many proteins were co-expressed with Fascin. For instance, Fascin level is positively correlated with ACSL4 and negatively correlated with ESR1. In addition, the ferroptosis signalling pathway was shown to be enriched by GSEA. Subsequently, we examined a panel of 10 breast cancer cell lines divided into two groups (Fascin high *vs* Fascin low levels) and observed that cells with high Fascin levels had an enhanced vulnerability to erastin-induced ferroptosis. In addition, our data from both gain- and loss-of-function studies showed that Fascin promotes ferroptosis. Taken together, these results may explain why TNBC is more sensitive to ferroptosis than non-TNBC subtypes.

Mechanistically, we observed that Fascin can downregulate xCT at the posttranscriptional level in TNBC. The results of previous studies indicated that Fascin is a downstream effector protein that is regulated by a number of other factors, such as transcription factors [34, 35], microRNAs [26, 36], and long-noncoding RNAs [15, 37]. However, accumulating evidence has demonstrated that Fascin can also regulate other factors. Liang et al. reported that Fascin regulates YAP/TEAD signalling by binding to the kinase MST1 in non-small-cell lung cancer [21]. Kang et al. discovered that Fascin directly interacts with MST2 and reduces its homodimerization in melanoma cells [22]. In another study, Liu et al. identified several Fascin-interacting proteins in laryngeal squamous cell carcinoma cells by IP followed by mass spectrometry. Through bioinformatics analysis, some crucial cellular processes, including cell adhesion, the regulation of protein ubiquitination, and small molecule metabolism, were shown to be associated with Fascin [23]. Therefore, Fascin may have much broader roles in cancers by binding potential partners. In the present study, we observed that Fascin directly interacts with xCT through endogenous IP. In addition, protein half-life and ubiquitination experiments further revealed that Fascin promotes the ubiquitin-mediated degradation of xCT. However, as Fascin is neither a ubiquitin ligase nor a deubiquitinase, the detailed mechanism by which Fascin regulates xCT stability remains to be further elucidated.

As mentioned above, xCT plays a significant role in the uptake of cystine required for GSH synthesis and is involved in ferroptosis. Although the basic functions and physiological roles of xCT have been observed in several studies, the underlying mechanisms of xCT regulation have yet to be fully elucidated, especially at the posttranslational modification level [38]. Our results provide additional evidence on the regulatory mechanisms of xCT and may provide a potential therapeutic target in breast cancer with high Fascin levels.

Pioneering works have demonstrated that therapy-resistant cancers are susceptible to ferroptosis [39, 40], leading us to research new treatment strategies to induce ferroptosis in cancer cells. Our previous studies revealed the occurrence of autophagy-mediated tamoxifen resistance in breast cancer [24] and the importance of crosstalk between autophagy and ferroptosis [41]. In the present study, we showed that MCF7/TAMR cells highly express Fascin and are more sensitive to erastin-induced ferroptosis. Suppression of Fascin with siRNA conferred MCF7/TAMR cells resistance to erastin-induced ferroptosis. In addition, proteomics analysis revealed that ferroptosis and the regulation of actin cytoskeleton signalling pathways are involved in MCF7/TAMR cell lines. Furthermore, erastin treatment effectively enhanced the effect of tamoxifen on MCF7/TAMR cells. These results indicate that Fascin is also involved in regulating ferroptosis in tamoxifen-resistant cancers. More importantly, drug-resistant cells may develop protective adaptations to conventional treatments, and inducing ferroptosis may be a promising treatment for tamoxifen-resistant cancers.

In summary, in the present study, we demonstrated that Fascin enhances the sensitivity of cells to ferroptosis by regulating xCT stability, and we propose the induction of ferroptosis in Fascin-high breast cancer cells as a potential therapeutic strategy.

## Supplementary information

Supplementary figure legends

Supplementary figure 1

Supplementary figure 2

Supplementary figure 3

Supplementary figure 4

Reproducibility checklist

## Data Availability

The raw data supporting the conclusions of this article will be made available by the corresponding author without undue reservation.
