# Supplementary material for: Fascin enhances the vulnerability of breast cancer to erastin-induced ferroptosis
Source: Cell Death Dis. 2022 Feb 14;13(2):150. doi: 10.1038/s41419-022-04579-1 (PMC8844358; doi:10.1038/s41419-022-04579-1)
Supplement: Supplementary file 1 — Supplementary figure legends [file 41419_2022_4579_MOESM1_ESM.docx]

**Supplementary figure legends**

Fig. **S1** IHC analysis of Fascin in TNBC patients and EMT markers upon Fascin knockdown cells.

**A** Four representative images from the IHC analysis of Fascin levels (negative, weak, moderate, and strong) obtained from TNBC patients. **B** Induction of EMT markers upon Fascin knockdown in HS578T cells. Cell lysates were collected after 72 h transfection.

Fig. **S2** Ferroptosis contributes to erastin-induced growth inhibition in HS578T and MDAMB231 cells.

HS578T and MDAMB231 cells were treated with erastin with or without the indicated inhibitors for 48 h and then assayed for cell viability. The data are displayed as the means ± s.d of three independent experiments.

Fig. **S3** xCT knockdown counteracted the Fascin knockdown-mediated resistance to ferroptosis in HS578T cells.

**A** Immunoblot analysis of Fascin, xCT, and β-actin in HS578T cells transfected with control or *Fascin* siRNAs with or without *xCT* siRNAs for 72 h. **B** HS578T cells transfected with control or *Fascin* siRNAs with or without *xCT* siRNAs were incubated with the indicated concentrations of erastin for 48 h and then assayed for cell viability. n.s. not significant, and ***p < 0.0001. The data are displayed as the means ± s.d of three independent experiments.

Fig. **S4** MCF7/TAMR cells were more resistant to tamoxifen than wild-type MCF7 cells.

MCF7 and MCF7/TAMR cells were incubated with the indicated concentrations of 4-OH tamoxifen for 48 h and then assayed for cell viability. The data are displayed as the means ± s.d of three independent experiments.
