# Supplementary figures and images for: Fascin enhances the vulnerability of breast cancer to erastin-induced ferroptosis
Source: Cell Death Dis. 2022 Feb 14;13(2):150. doi: 10.1038/s41419-022-04579-1 (PMC8844358; doi:10.1038/s41419-022-04579-1)

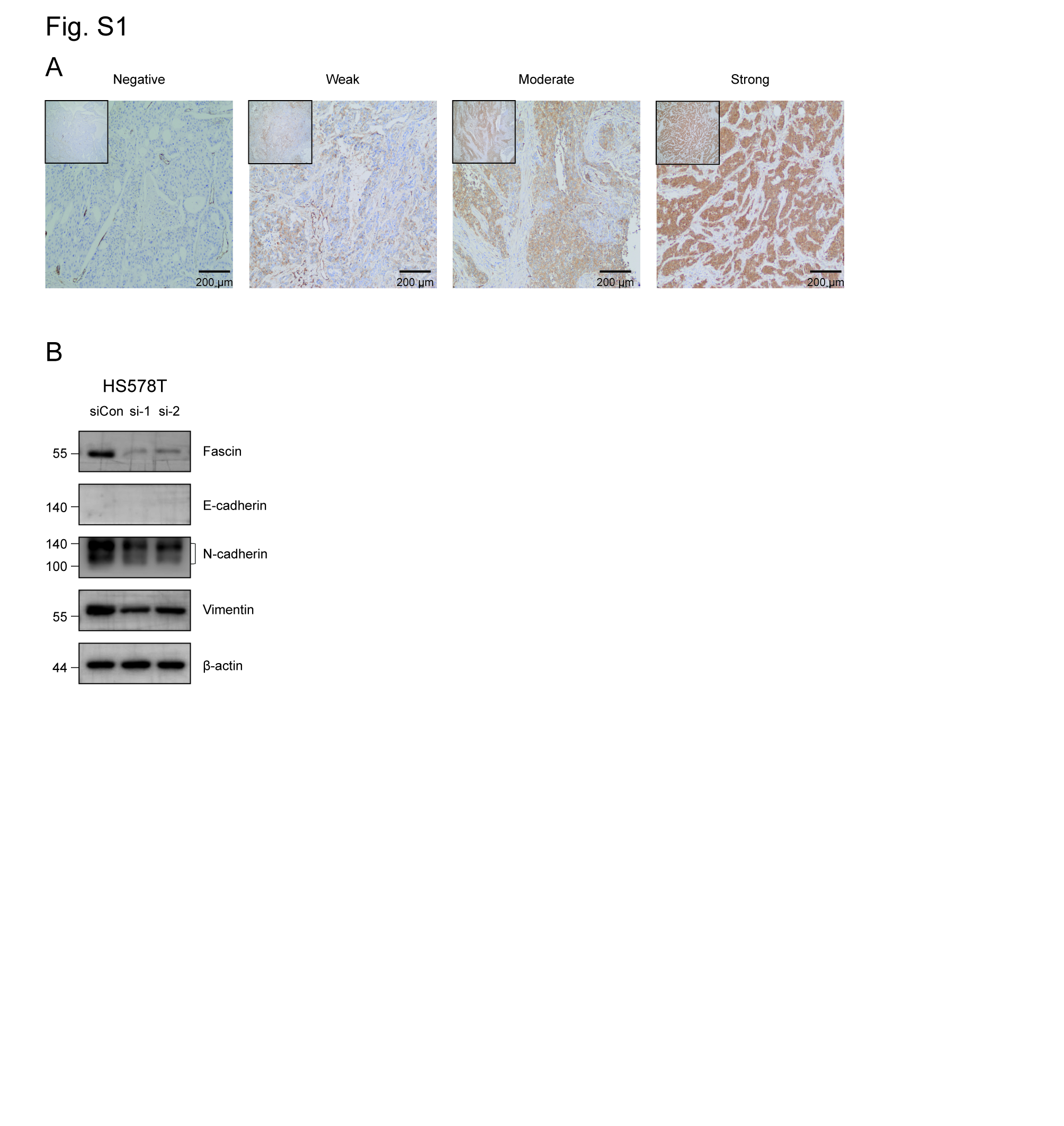

Supplement: Supplementary file 2 — Supplementary figure 1 [file 41419_2022_4579_MOESM2_ESM.tif]

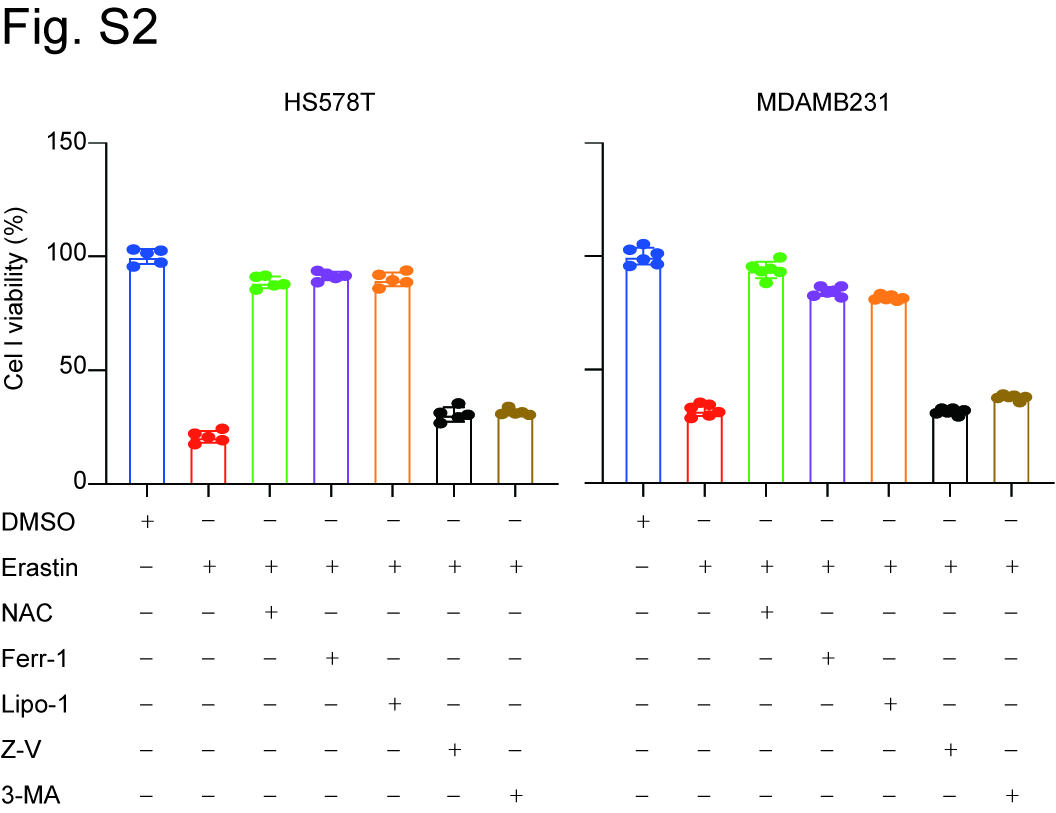

Supplement: Supplementary file 3 — Supplementary figure 2 [file 41419_2022_4579_MOESM3_ESM.tif]

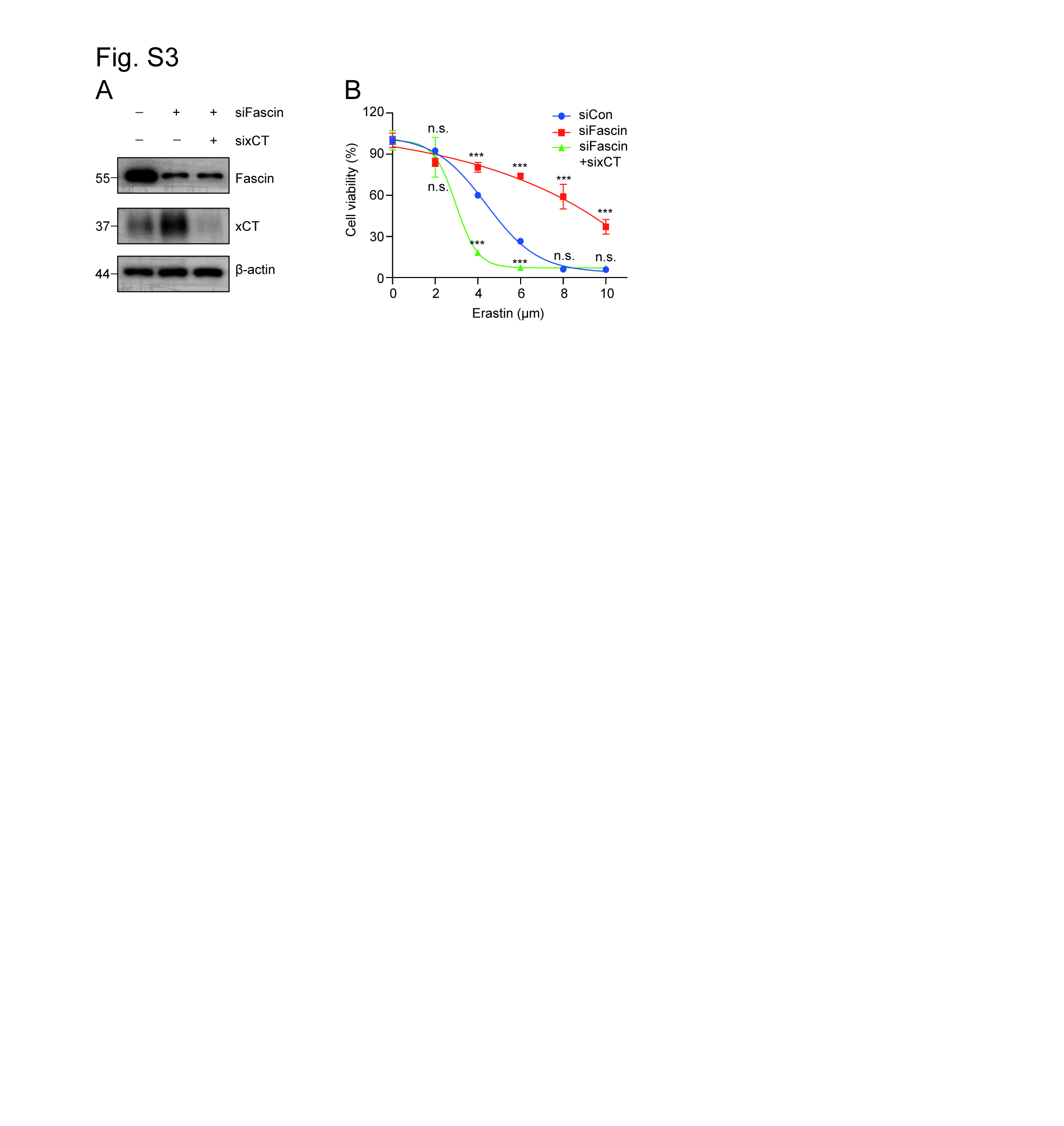

Supplement: Supplementary file 4 — Supplementary figure 3 [file 41419_2022_4579_MOESM4_ESM.tif]

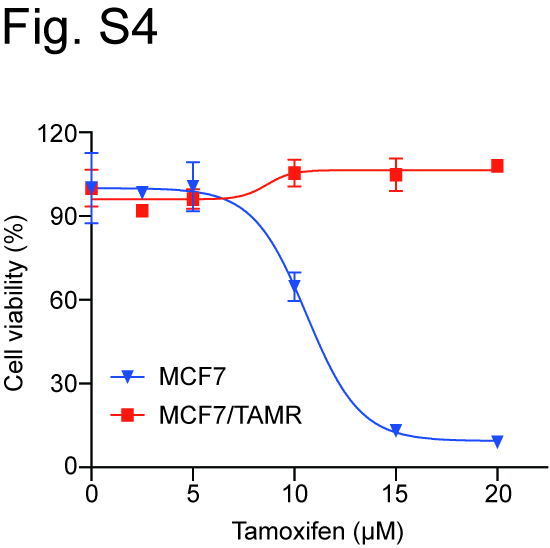

Supplement: Supplementary file 5 — Supplementary figure 4 [file 41419_2022_4579_MOESM5_ESM.tif]
